# Conservative Management of Madelung Deformity of the Wrist in a 15-Year-Old Male: A Case Report

**DOI:** 10.7759/cureus.49594

**Published:** 2023-11-28

**Authors:** David Savage-Lobeck, Sujan Gogu

**Affiliations:** 1 School of Medicine, St. James School of Medicine, Chicago, USA; 2 Sports Medicine, South Texas Health System, Edinburg, USA

**Keywords:** pediatric imaging, pediatric sports medicine, madelung deformity, conservative medical management, madelung’s deformity

## Abstract

A 15-year-old boy presented to an outpatient sports medicine clinic with mysterious wrist pain for three months. Imaging diagnosed this mysterious wrist pain as a Madelung deformity, a rare condition characterized primarily by the early cessation of growth in the volar-ulnar distal physis of the radius. Patients may present with restricted wrist range of motion, pain, and ulnar tenderness, but they may also be asymptomatic. Traditionally, treatment is surgical, but this case was managed with conservative measures primarily, with improvements in both function and outcomes. This case report should serve as a reminder to include rare diagnoses in the differential when working up joint pain and to show the role of conservative management in Madelung deformity.

## Introduction

First documented in academia by Otto Madelung in 1878 [1], the Madelung deformity of the wrist is currently described as the following: It most commonly presents in adolescents aged 8 to 14, is often bilaterally seen [2,3], and occurs most commonly in females (4:1 predominance) [4]. Radiologically, this malformation is characterized by a palmar and ulnar inclination beginning at the distal articular surface of the radius, with concomitant dorsal subluxation of the distal ulna [3]. Vickers previously described the etiology of the deformity as arising from the distal radius next to the physis and inserting into the lunate, known as a Vickers ligament [4]. Current thought follows that this ligament restricts ulnar growth in the palmar segment of the distal segment of the radius. Vender and Watson previously classified Madelung/Madelung-like deformities into four categories: idiopathic, post-traumatic (such as the repetitive micro-trauma experienced by gymnasts [5]), dysplasical (such as in dyschondrosteosis, a condition that commonly presents with Madelung deformities), or genetic (Turner syndrome). Their study also suggested that past medical history is critical in distinguishing deformity etiology as well as physical findings such as unilaterality of deformity and a less overall severe deformation [6]. Radiologic differentiation between the two is defined as the presence or absence of the Vicker’s ligament, which is only present in congenital (or “true”) Madelung deformities [7].

## Case presentation

A 15-year-old male presented to our clinic with a complaint of dull left wrist pain for three months. He cited no immediate preceding infection, prior injury, relevant family history, or similar symptoms in the past. He noted that he recently began catching for his baseball team, an activity he performs with his left hand, and the pain began the day after his first game, which was the day before his pain started. Besides catching some hard pitches, he cited no hard falls or other inciting trauma. This pain did not radiate from the wrist area at the time of the examination. The patient had only taken ibuprofen with slight relief before his visit to the clinic. He had previously visited a freestanding ER, but the plain radiographs taken there were read as normal.

A physical exam showed a full range of motion (ROM) of the shoulder and elbow. Wrist ROM was reduced, but the patient reported this was mostly limited by pain. The patient's digital movement had full ROM. Forearm tenderness was noted on and immediately around the left ulnar styloid. A small, tender, firm nodule was palpable at this point, which was slightly compressible into the wrist with a rebound to its original position. Circulation and sensation were normal in the limb.

**Figure 1:**
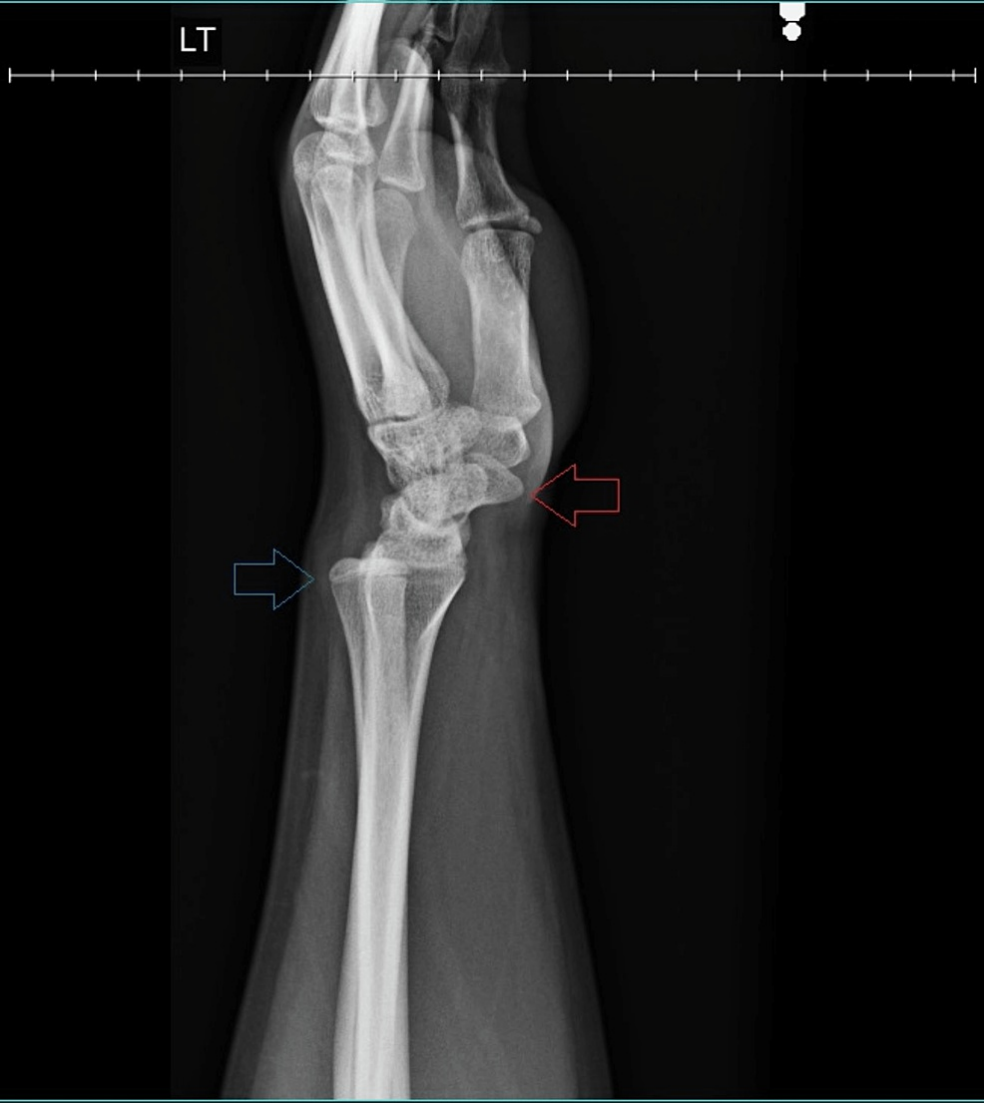
Lateral X-ray of the wrist joint. The distal ulna has undergone dorsal subluxation (blue arrow), and the hand is subluxated volarly (red arrow).

**Figure 2:**
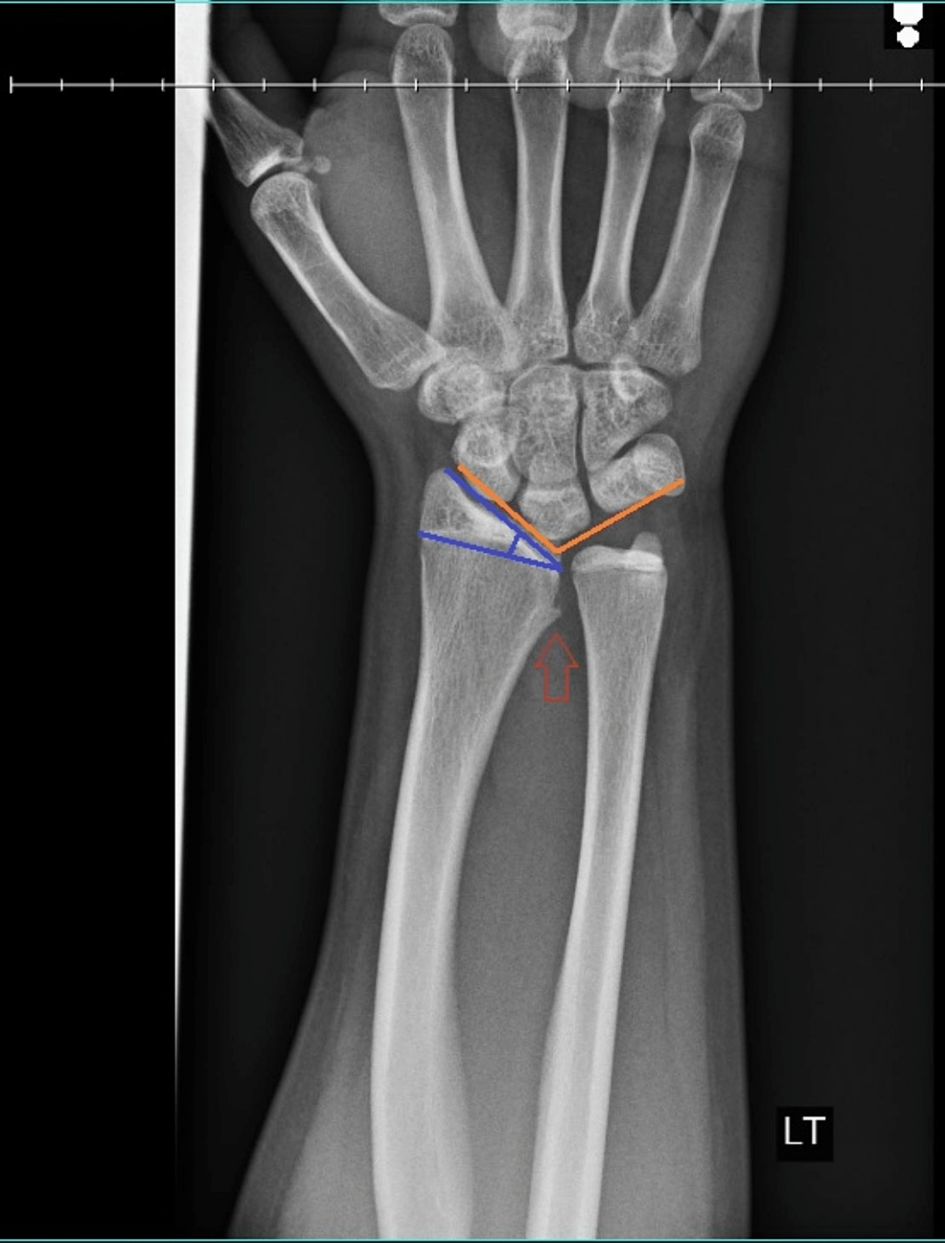
Anteroposterior X-ray. The joint angle of the carpus is approximated based on current positioning (orange lines). The radius has an increased inclination angle (blue lines). A spur is noted on the medial surface of the radius (red arrow).

X-rays taken in the clinic subjectively appeared normal to physician interpretation, with only a slight spur on the interior surface of the radius and a very slight opening of the radioulnar joint noted. The lateral view was significant for gross posterior displacement of the distal ulna relative to the wrist. The ulnar dislocation was suspected; the closed reduction was attempted at this time without success. The plain radiographs can be seen in Figures 1, 2.

**Figure 3:**
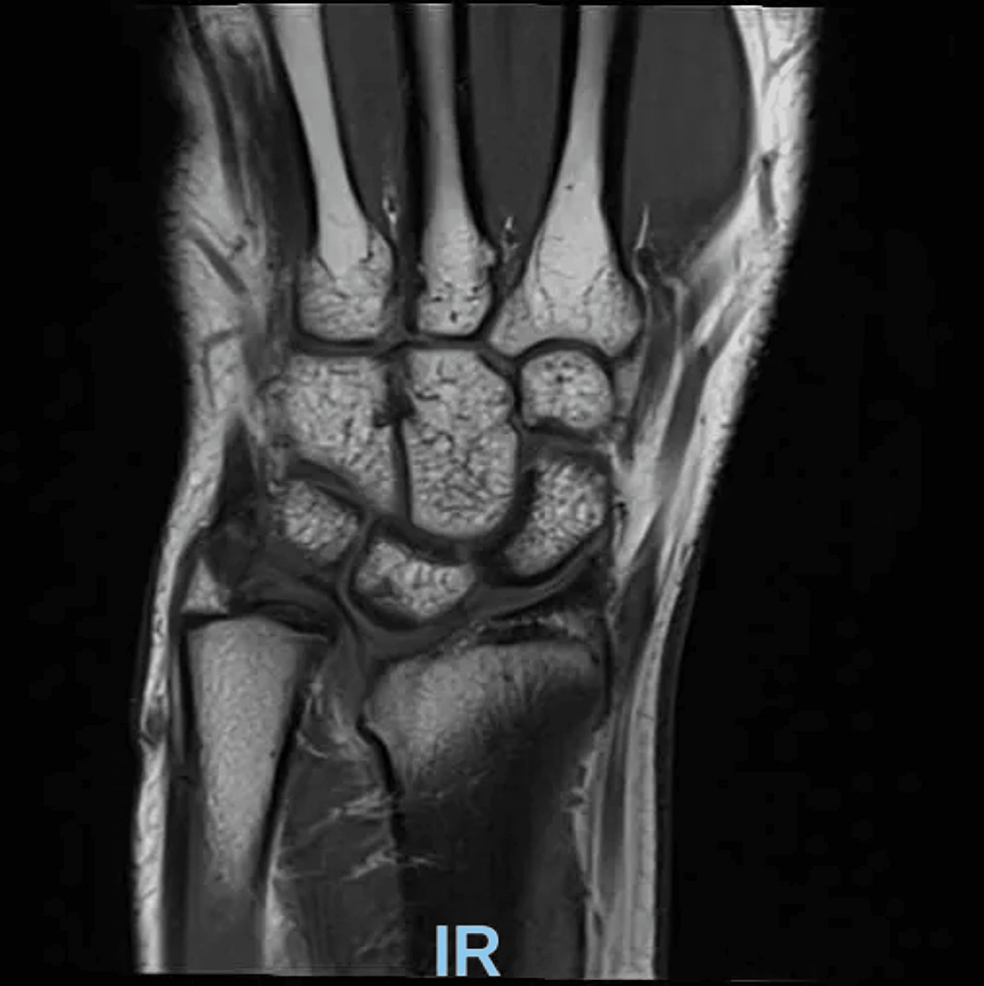
MRI of the wrist joint. The radial physis is clearly visualized, and the volar-lateral physis is notably absent, indicating premature closure. The articular cartilage displays an elongated configuration. Chronic tears of the foveal and ulnar attachments to the TFCC are noted dorsally.

**Figure 4:**
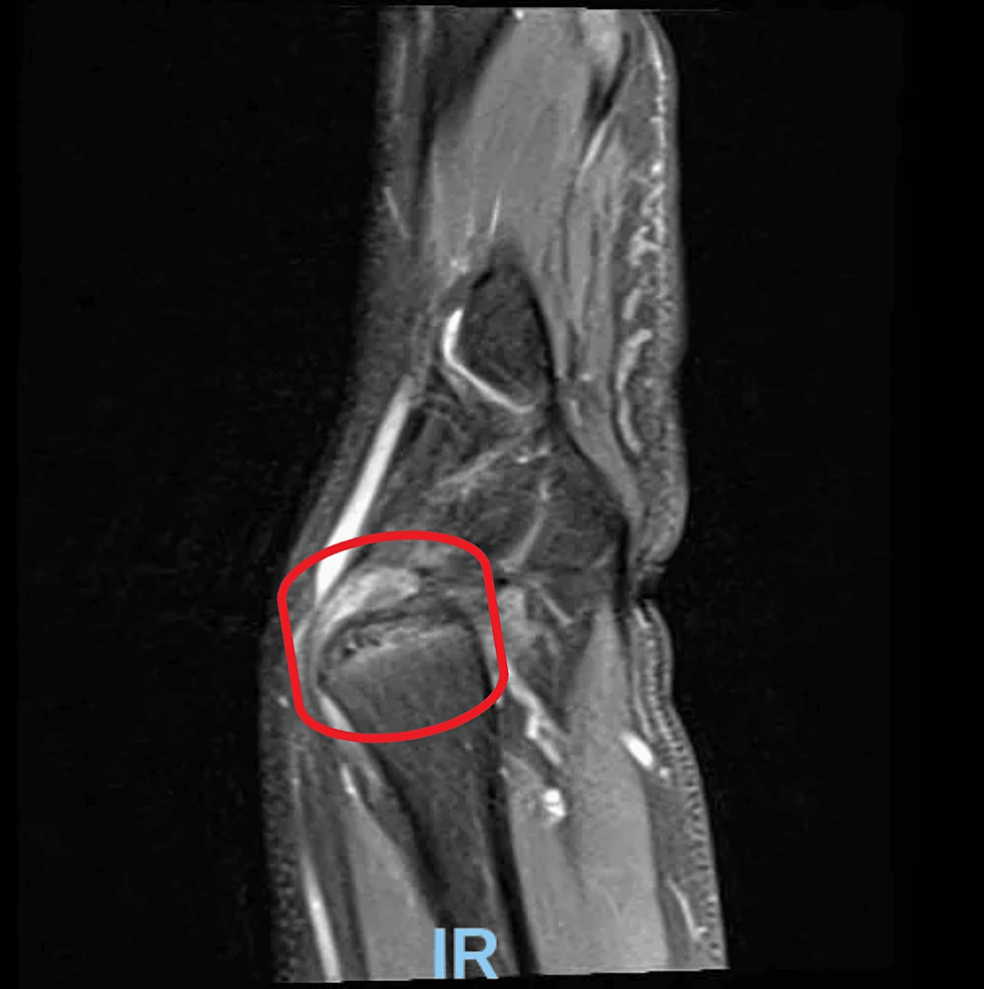
MRI of the wrist joint. Positive ulnar variance with marrow edema at the ulnar head is seen (red oval).

**Figure 5:**
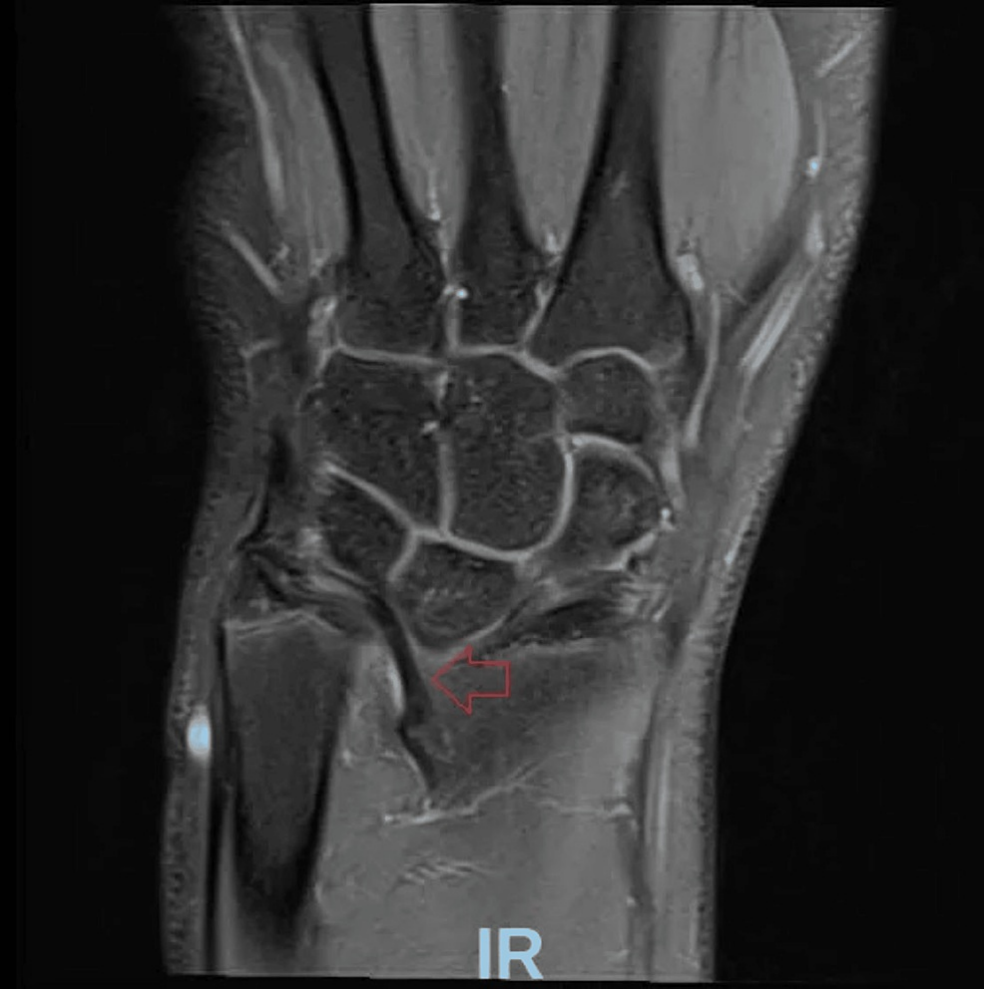
MRI of the left wrist. Here, a Vicker’s ligament is visualized (red arrow), originating from the metaphysis of the distal radius. There are findings consistent with dysplasia of the radial epiphysis. Increased volar tilt and radial inclination are noted.

The patient was referred to outpatient magnetic resonance imaging (MRI) for assessment of possible ligament damage to the triangular fibrocartilage complex (TFCC) and was given precautions against strenuous physical activity. The MRI revealed findings consistent with a likely Madelung deformity; images showing these findings can be seen in Figures 3-5.

The patient returned to the clinic for consultation and interpretation of the MRI results; upon further physical examination, the deformity was found to be bilateral in nature, which was not noticed prior. Neither wrist was symptomatic at this time. A trial of combination physical and occupational therapy, with an emphasis on range of motion, was initiated for six weeks, with continuation for another six weeks after improvement was noted. The patient continued ibuprofen as needed, and no additional analgesics were prescribed. Protective measures for increased activity were implemented, such as core training and wrist taping, and four months after initial contact, the patient was advised to continue with conservative therapy indefinitely until reassessment. After several clinical visits, a full and eventual return to full sport activity participation was planned.

## Discussion

In comparison to the majority of previously reported cases of congenital Madelung deformity, the case we observed arose suddenly after the onset of strenuous physical activity. It combines aspects of both clear-cut, slowly worsening Madelung deformity [1] and Madelung deformity due to high-stress activity [6]. Radiologically, the case is easily diagnosed when advance imaging is deployed; however, in a primary care setting, mistaking a Madelung deformity for an ulnar dislocation is an easy pitfall. Signs on a plain radiograph that may help in accurate diagnostics include radial bowing, a visible volar shift of the ulnar head, an increased inclination angle of the radius, and a V-shaped carpal joint angle [4]. 

In this case, the patient's condition was managed conservatively, improving overall wrist function and decreasing pain with combined physical and occupational therapy; this is consistent with prior cases of conservative management in patients with Madelung deformity [1]. While some studies show that prophylactic release of the ligament via a Langenskiöld procedure may have helped control or prevent pain in patients with Vicker's ligament [7], there is not extensive evidence to conclude it would have been of definite benefit in this case. Despite not pursuing surgical treatment, the patient’s level of athletic functioning improved compared to his prior level; while this does not necessarily mean he will not require surgical intervention in the future, the improvement seen with the conservative solutions implemented is a positive prognostic sign for his sports career. 

It is important to note that the diagnosis of Madelung deformity was not suggested until advanced soft-tissue imaging was performed and the Vicker's ligament was clearly visualized. There are a number of potential reasons as to why the condition was not mentioned prior in the differential diagnoses, but the most likely offenders are the rarity of the condition in similar patients (the prevalence is.03% in the general population [2], with a 4:1 female predominance [3]) and the lack of the condition's prevalence in medical literature. While the former is unlikely to change, the latter can be resolved with education and research for first-line caregivers. We propose that it would be beneficial to patients if a list of advanced differential diagnoses for wrist pain were kept on hand in primary care clinics. 

Limits of this trial of conservative management include, primarily, the lack of a varied study population, which is mainly due to the rarity of the condition (estimated to be present in 0.03% of the general population [2], with this increasing to 10% in patients with Turner's syndrome [8]). This lack of prevalence often results in varied methods of attempting to resolve any pain or other symptoms and could potentially be resolved by conducting a long-term trial in a larger population. A major strength of this study is the frequency of clinical updates, which may not be possible in a larger population due to logistical challenges. Care should be taken to balance the two should a larger trial of similar treatment be attempted in the future. 

## Conclusions

Madelung’s deformity, while rare, is a diagnosis that should not be forgotten on initial clinic encounters for wrist pain with associated deformity. Surgical intervention, while commonly used to resolve cases of Madelung’s deformity with the presence of Vicker’s ligament, is not totally necessary to improve function, quality of life, or long-term outcomes; conservative management, including but not limited to physical and occupational therapy, should be considered in similar cases as the one presented here.
